# Extensive Cutaneous Mastocytosis After Pregnancy

**DOI:** 10.7759/cureus.7057

**Published:** 2020-02-20

**Authors:** Eric Fris, Jaime Tschen

**Affiliations:** 1 Dermatology, McGovern Medical School, Houston, USA; 2 Dermatology, St. Joseph Dermatopathology, Houston, USA

**Keywords:** mastocytosis, pregnancy, tmep

## Abstract

Mastocytosis is an uncommon disease involving the proliferation of mast cells within at least one organ system, most commonly the skin. One rare variant is telangiectasia macularis eruptive perstans (TMEP). The telangiectatic tan-brown macules are highly characteristic, although a biopsy is indicated to confirm the diagnosis. We present a 33-year-old white woman who presented for a skin check with concern for a four- to six-year history of "moles" present on the majority of body surface area. Her lesions presented shortly after her first pregnancy and spared sun-exposed face, neck, and extremities. Both of these features are rather unusual in TMEP. In this asymptomatic patient, workup focused on excluding systemic manifestations and discussion of cosmetic treatments. Punch biopsies revealed nests of CD117+ mast cells as well as increased basal melanocytes. Because the lesions spared sun-exposed regions, sunbathing was advised for initial treatment.

## Introduction

Mastocytosis is a rare disease characterized by tan-brown macules often diffusely spread across multiple regions of the skin. Patients usually present with mild-to-moderate pruritis and complaints about their appearance. The skin manifestation is the most common presenting sign of the widespread proliferation of mast cells in at least one tissue type. In this report, we will present a rare variant of this disease-telangiectasia macularis eruptive perstans (TMEP)-as well as the characteristics of this patient.

## Case presentation

History

A 33-year-old white woman presented for a skin check with concern for moles present on the majority of body surface area. She states that they first presented four to six years ago, shortly after her first pregnancy, and the lesions are asymptomatic. She is a non-smoker with no significant family history. Her thyroid was normal, and her medical history is otherwise non-contributory.

Physical exam

**Figure 1:**
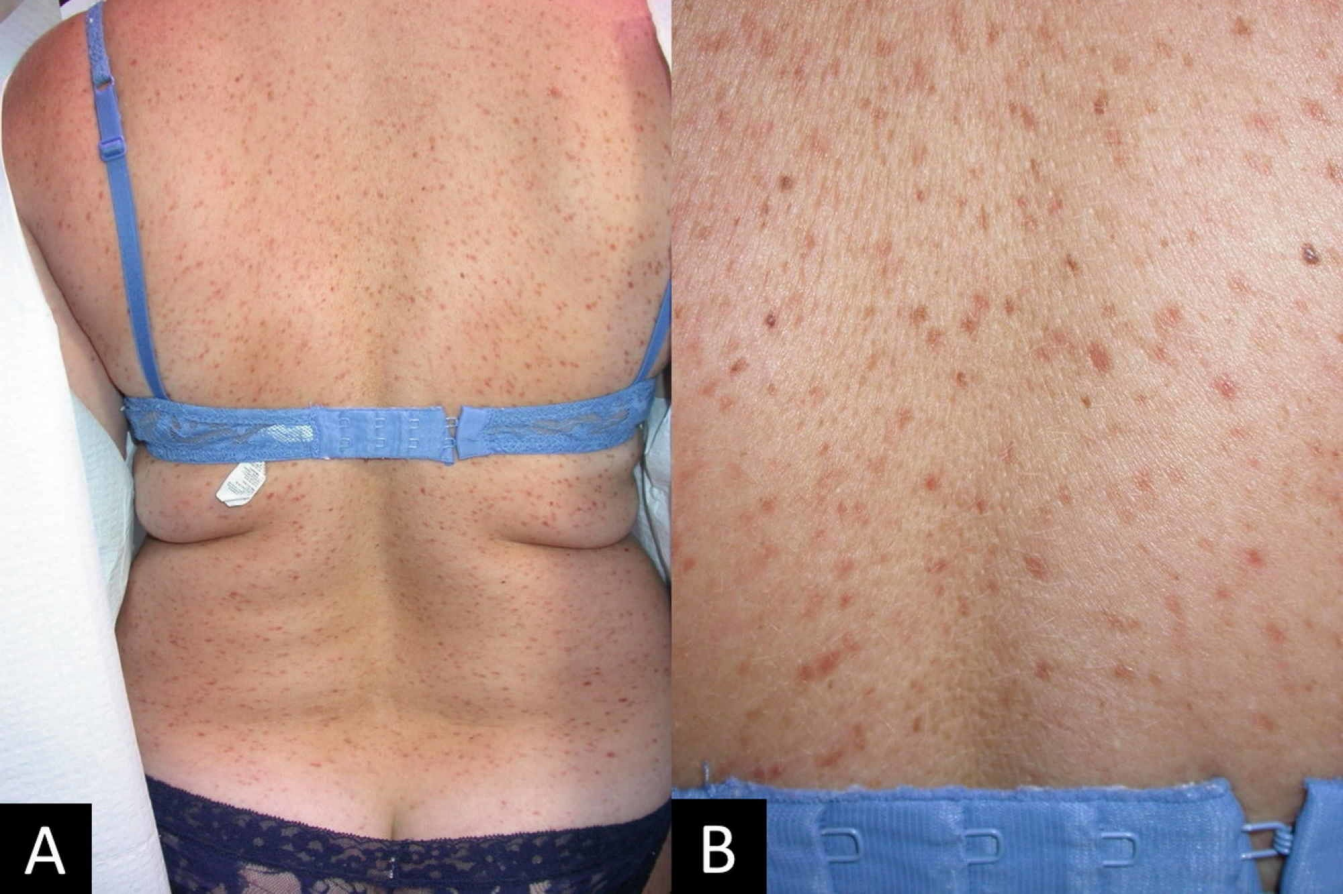
(A) Multiple non-coalescing brown macules on the upper and lower back. (B) Closer view of the skin findings in detail.

**Figure 2:**
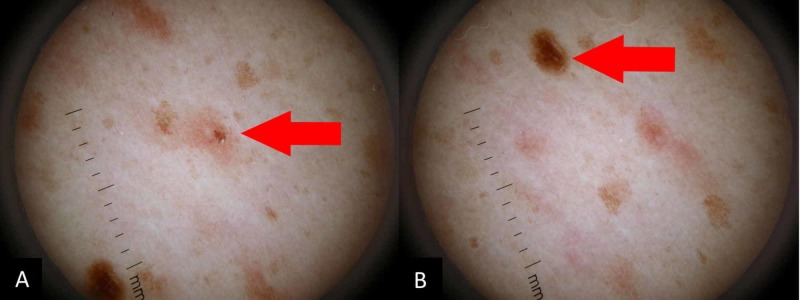
(A) Dermatoscopic evaluation of a mildly pigmented macule with poorly defined margins. (B) Dermatoscopic evaluation of a deeply pigmented macule with sharper margins.

On exam, the patient has diffuse presentation of asymptomatic, dark, macules sparing the face, neck, genitalia, and sun-exposed areas of limbs (Figure 1A, 1B). The macules were not pruritic or tender. In Figure 2A, 2B, the macules can be better appreciated under the dermatoscope.

 

Histopathology

**Figure 3:**
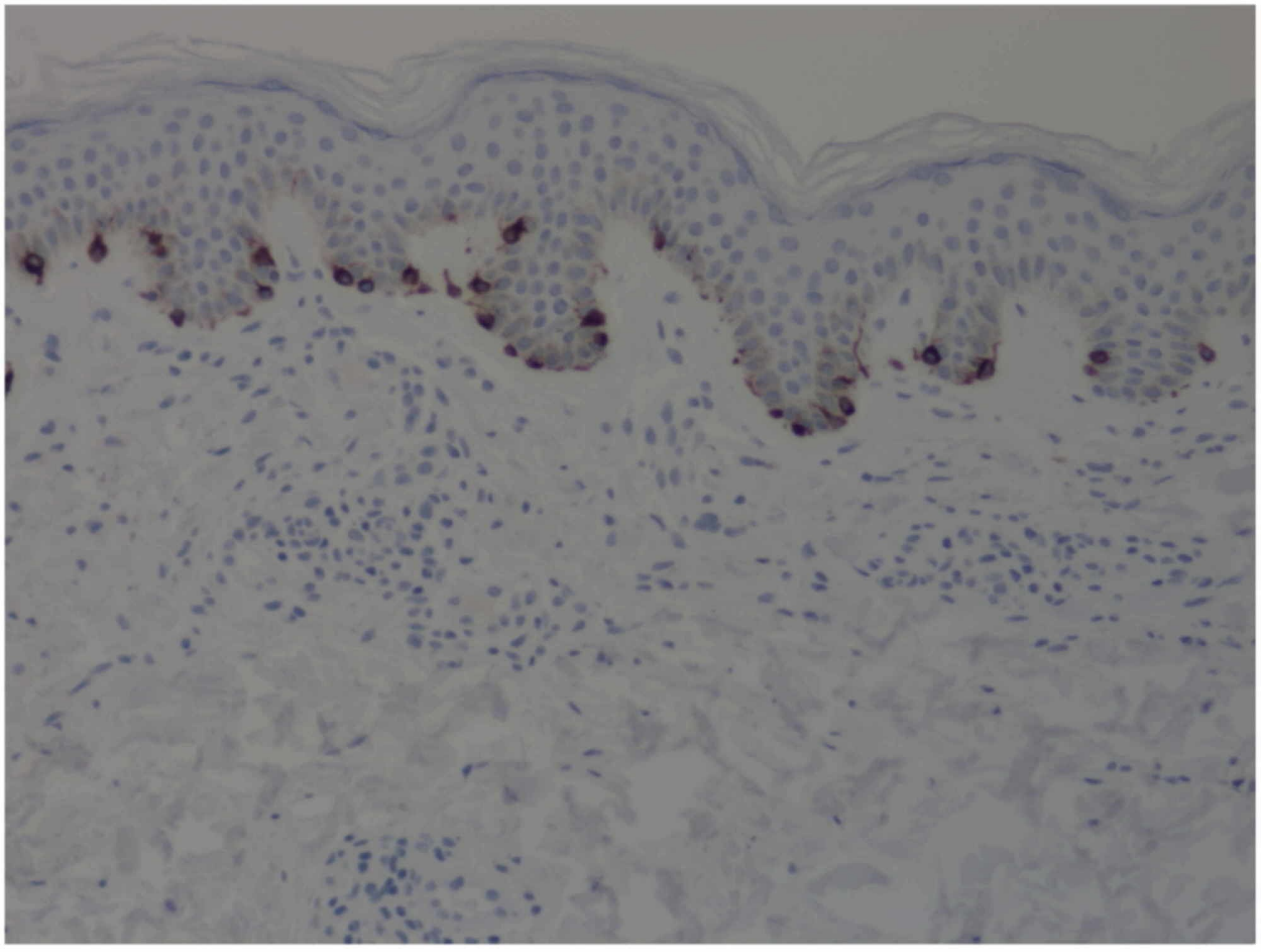
CD117+ stain showing mast cells in the dermal layer.

**Figure 4:**
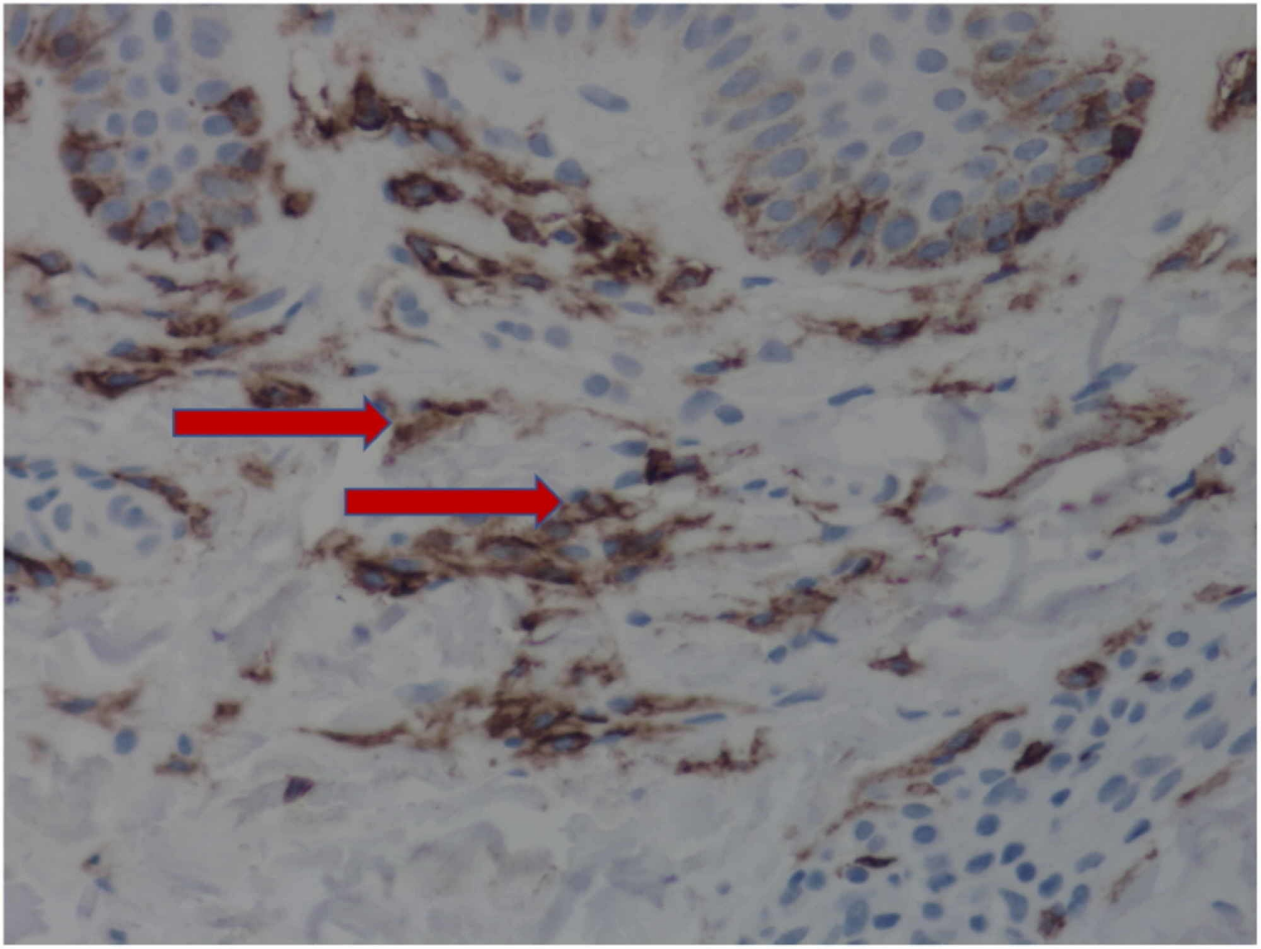
Higher power magnification reveals nests of mast cells (arrows).

Two 2-mm punch biopsies revealed markedly increased numbers of mast cells in the superficial dermis. Hyperpigmentation of the basal layer, scattered lymphocytes, and eosinophils are also seen. Special stains with appropriate controls show numerous (more than 100) mast cells with CD117 and some increased numbers of basal melanocytes (Figures 3, 4).

Assessment

Sunbathing was advised. The patient refused further workup and has not returned for follow-up.

## Discussion

Mastocytosis is an uncommon condition caused by the abnormal accumulation of mast cells in at least one organ system, most commonly the skin [1]. TMEP is a rare variant of mastocytosis, usually consisting of telangiectatic tan-brown macules [2]. The lesions tend not to cause urticaria, nor does TMEP usually present with constitutional symptoms [3]. However, induction of mast cell degranulation can induce symptoms, and triggers such as known immunogenic stimuli and certain medications should be avoided [1].

Treatment is highly subject to the individual patient’s symptoms and triggering events. There is no gold standard, and the literature on treatment remains sparse given that most presentations are asymptomatic. In this case, sunbathing was advised as a first-line treatment because the patient’s sun-exposed limbs were lesion-free. Antihistamines would be suggested if the patient experienced urticarial symptoms. Other pharmacologic mediators in the mast cell-leukotriene pathway are sometimes used as well for symptomatic relief [1]. In some cases, phototherapy is efficacious and is an alternative to pharmacologic intervention. Psoralen and ultraviolet A radiation (PUVA) is beneficial in the short term but provides only temporary management [4]. Pulse therapy at 585 nm wavelength has demonstrated efficacy in two studies, with recurrence at 14 months post-treatment [5].

## Conclusions

TMEP has a highly variable presentation. Therefore, response to treatment is correspondingly variable. A thorough history and physical examination is important to determine an escalating treatment plan. A final treatment plan will be guided by the nature of symptoms, cosmetic concerns, and response to escalating therapies. If sun-exposed regions are free of lesions, as in this case, sunbathing is recommended prior to initiating more costly treatment options (e.g. PUVA). Ultimately, the patient should be counseled that no single treatment is likely to be completely curative over the long term, although PUVA and pulse therapy at 585 nm have short-term efficacy.
